# S218. INSIGHT AND SUBJECTIVITY

**DOI:** 10.1093/schbul/sby018.1005

**Published:** 2018-04-01

**Authors:** Clara Monserrat, Dolores Sanchez-Cabezudo, Carles Garcia-Ribera, Carmina Massons, Lourdes Nieto, Esther Pousa, Jesús Cobo, Ada Ruiz, Susana Ochoa, Judith Usall

**Affiliations:** 1 Hospital del Mar de Barcelona; 2 Hôpital Philippe Pinel, Amiens; 3 Hospital de Sant Pau de Barcelona; 4 Corporació Sanitària i Universitaria Parc Taulí, Sabadell; 5 Instituto Nacional de Psiquiatría Ramón de la Fuente Muñiz, México D. F; 6 Sant Joan de Déu de Barcelona

## Abstract

**Background:**

The awareness of mental disorder or insight refers to the ability to perceive the disorder itself and the symptoms, the effects of the treatment and the social consequences of the disorder; and also the ability to attribute the symptoms to a mental disorder. Lack of insight is frequent in schizophrenia and is associated with a low adherence to the treatment and to a worse evolution. A greater insight has been associated with a lower psychopathological severity and with higher levels of depression.

On the other hand, subjective insight refers not only to what happens to the patient but also to how he feels and to the perception of the changes that he undergoes during the psychotic experience. The subjective perception of change is a position that can easily lead to connect with painful and depressive feelings, so it can be assumed that subjective insight could be related more consistently with the depressive symptoms than the clinical insight.

**Methods:**

Observational cross-sectional study of a group of 114 schizophrenia patients treated in the psychiatry devices of the Parc de Salut Mar and Parc Taulí Instruments: SUMD, Markova and Berrios Scale and Calgary scale for depression in psychosis.

**Results:**

Subjective insight is significantly correlated with Lindenmayer’s depressive factor and depression level measured by a Calgary scale.

Clinical insight correlates with positive and excitatory symptoms. The time of evolution explains the non-awareness of the social consequences of the disease.

**Discussion:**

The subjective insight into schizophrenia is mainly related to the depressive symptoms. The clinical insight into schizophrenia is related to positive symptoms.

